# Strengthening the quality of paediatric primary care: protocol for the process evaluation of a health systems intervention in South Africa

**DOI:** 10.1136/bmjgh-2018-000945

**Published:** 2018-10-23

**Authors:** Jamie Murdoch, Robyn Curran, Max Bachmann, Eric Bateman, Ruth Vania Cornick, Tanya Doherty, Sandra Claire Picken, Makhosazana Lungile Simelane, Lara Fairall

**Affiliations:** 1 School of Health Sciences, University of East Anglia, Norwich, UK; 2 Knowledge Translation Unit, University of Cape Town Lung Institute, Cape Town, South Africa; 3 Norwich Medical School, University of East Anglia, Norwich, UK; 4 Health Systems Research Unit, South African Medical Research Council, Cape Town, South Africa

**Keywords:** child health, health systems evaluation, paediatrics, prevention strategies, other study design

## Abstract

**Background:**

Despite significant reductions in mortality, preventable and treatable conditions remain the leading causes of death in children under five within South Africa. The WHO’s Integrated Management of Childhood Illness (IMCI) programme has been widely implemented to address the most common causes of mortality in children under five. Although effective, limitations in IMCI scope and adherence have emerged. The Practical Approach to Care Kit (PACK) Child guide has been developed to expand on IMCI and address these limitations. It is intended as a clinical decision support tool for health workers with additional systems strengthening components, including active implementation and training strategy to address contextual and organisational factors hindering quality of care for children. Implementation is complex, requiring comprehensive pilot and process evaluation. The PACK Child pilot and feasibility study will sample 10 primary care facilities in the Western Cape Province. Staff will be trained to integrate the PACK Child guide into routine practice. The process evaluation will investigate implementation and health systems components to establish how to optimise delivery, strengthen IMCI principles and factors required to support effective and sustained uptake into everyday practice.

**Methods:**

Mixed method process evaluation. Qualitative data include interviews with managers, staff, caregivers and policymakers; observations of training, consultations and clinic flow. Quantitative data include training logs and staff questionnaires. Quantitative and qualitative analysis will be integrated to describe study sites and develop explanations for implementation variation.

**Discussion:**

The process evaluation will provide the opportunity to document implementation and refine the programme prior to a larger pragmatic trial or scale-up.

**Summary box**

- The WHO’s Integrated Management of Childhood Illness (IMCI) has been widely implemented in South Africa.
- Despite improving quality of care, preventable and treatable illness remain leading causes of death in children under five.
- The Practical Approach to Care Kit (PACK) Child guide was developed to address the limitations of IMCI, using a clinical decision support tool, training package and systems strengthening intervention.
- Implementation of PACK Child is complex, requiring a detailed process evaluation to understand how to support effective and sustained uptake within clinical practice.

## Background

In South Africa, the management of common childhood illnesses at a primary healthcare level remains poor with preventable and treatable conditions, particularly pneumonia and diarrhoea, remaining the leading causes of death in children under five.^1^ Although the under-five mortality rate has declined over the past decade, largely due to the high coverage of the prevention of mother to child HIV transmission programme, by 2015, South Africa had not reached the Millennium Development Goal 4 to reduce child mortality by two thirds, with an under-five mortality rate of 42 per 1000 live births.^2^ Considerable ongoing improvements in health worker skills and quality of care are required to reach the Sustainable Development Goal target of an under-five mortality rate of at least 25 per 1000 live births by 2030.

The WHO’s Integrated Management of Childhood Illness strategy (IMCI)^3^ was developed to address the top causes of mortality in children under five and is the standard of care in over 100 low-income and middle-income countries, including South Africa.^4^ A multicountry review of IMCI^5^ confirmed improvements in prescription accuracy, treatment and health service quality, and a 2016 Cochrane review^6^ found evidence of a reduction in neonatal and infant mortality. However, an evaluation of IMCI’s impact since its introduction in 1998 reported variable adherence to the strategy’s guidance.^7^ In many low-resource settings, IMCI is implemented as one-off training of primary care workers (usually nurses), with little ongoing supervision and infrequent updating.^8^ Furthermore, the IMCI strategy does not address the health needs of children over 5 years, those with conditions needing regular follow-up and guidance is limited for children attending for vaccination or routine consultations. There is therefore a need for a different approach to building the skills and capacity of frontline health workers providing care for children at a primary healthcare level.

To address these gaps, the Knowledge Translation Unit (KTU) has developed a paediatric version of its Practical Approach to Care Kit (PACK),^9^ intervention, which applies some IMCI principles while addressing several shortcomings in provision of primary care to children. PACK Child is modelled on and complements PACK Adult, which was trialled and scaled up in South Africa to over 30 000 clinicians in more than 3500 clinics.^10^ ^11^ It is underpinned by an ethos to support, motivate and enable healthcare providers to tackle multiple coexisting acute and chronic childhood conditions. The intervention includes the PACK Child guide, staff training and also systems strengthening and enhanced supervision with regular updates as guidance and policies change. Successful implementation of PACK Child is likely to be complex, requiring detailed evaluation of the ability of an ‘adult-inspired’ programme to change the scope of clinicians to deliver paediatric primary care and whether PACK Child will augment or undermine other priorities like early childhood development and preventive care. This paper describes the protocol for a process evaluation of the first PACK Child pilot in the Western Cape Province, which will provide important information regarding the feasibility of the intervention, alignment with IMCI and factors influencing future wide scale uptake of the guide into routine practice.

### PACK Child intervention

The PACK Child guide covers 63 symptoms and 16 long-term conditions most commonly seen in primary care as well as an approach to screening the well child. It is intended for use in consultations with children 0–13 years and is aligned to recognised standards for guideline development.^12 13^

**Figure 1:**
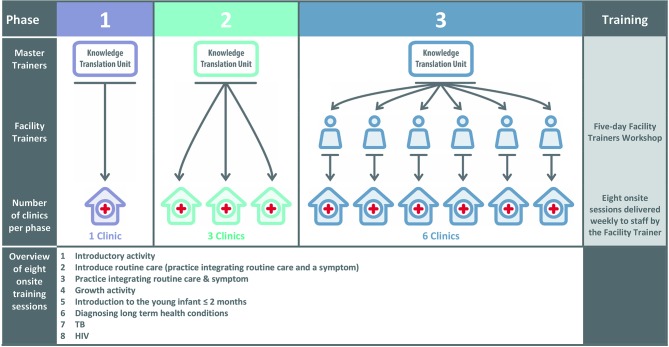
PACK Child training programme and cascade model.

Following the educational outreach model of PACK Adult (R Cornick, submitted in this collection). PACK Child is designed to support large-scale implementation with onsite training and task-sharing, increasing the capacity of clinicians to deliver paediatric primary care. The model of PACK Child training sessions^14^ involves identifying priority content, selecting key messages and developing a curriculum of cases to convey these messages during a programme of eight onsite training sessions, each lasting between 1½ and 2 hours (see figure 1). The approach includes training usual health department staff as onsite trainers to ensure effective delivery and scalability. The training also emphasises the alignment of the PACK Child content to IMCI, the integration of care for the child’s carer using PACK Adult and upskilling of all clinical staff to encourage a multidisciplinary approach to primary paediatric care.

### Process evaluation

Process evaluations typically evaluate how and whether interventions are delivered as intended and whether such implementation is congruent with the theory underpinning the intervention.^15–17^ A principal task lies in evaluating the extent to which resources and activities supporting the intervention function to deliver intended outputs, with subsequent improvements in outcomes. For the PACK Child study, process evaluation is required into how the training sessions, PACK Child guide and systems strengthening activities (study inputs), function to enable, motivate and support (mechanisms of action), an increased scope of practice for healthcare providers (outputs), with subsequent improvements in the management of acute and chronic child conditions (outcomes). A central focus also lies in identifying contextually relevant strategies for successful implementation, practical difficulties in adoption, delivery and maintenance to inform wider implementation.

The overall aim of the process evaluation is to examine how PACK Child is implemented within primary care facilities in order to refine the intervention prior to testing in a pragmatic trial and to identify key processes and outcomes to measure in the trial.

The research objectives are to determine:

1. The impact of the organisational and social contexts of PACK Child training as it is delivered and embedded into routine practice.
2. The fidelity of intervention delivery.
3. Staff perspectives of delivery and participation in the training intervention.
4. Carer’s (and children as appropriate) perspectives of how PACK Child consultations meet carer and child needs.
5. How to optimise delivery of PACK Child within routine practice.
6. Which components of the PACK Child intervention affect which change in healthcare processes and outcomes.
7. Barriers to and facilitators of change through implementation of PACK Child guide in primary care.

## Methods

### Design considerations

Variation in implementation of PACK Child may arise due to differences in delivery and receipt of intervention components, differing contexts of facilities and because many different individuals will be involved in delivery. Process evaluation design, like healthcare interventions, therefore requires a theoretical framework to structure the evaluation across multiple sites. In designing the process evaluation for piloting PACK Child, we are drawing on Bronfenbrenner’s ecological model of behaviour,^18 19^ to conceptualise healthcare interventions as events that disrupt complex social systems,^20^ operating across multiple contextual levels.

**Figure 2:**
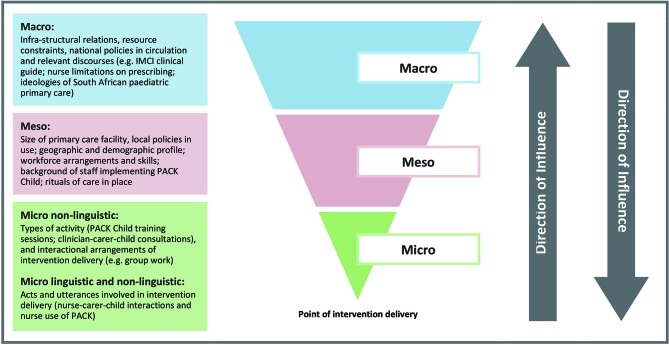
Investigating PACK Child within context (Murdoch, 2016).

The process evaluation will adopt a linguistic ethnographic^21 22^ methodology, which combines strengths of linguistics and ethnography to systematically investigate human behaviour within context. Linguistic ethnography has been described as a site of encounter for different disciplines to help resolve common difficulties in the analysis of social action.^22^ It provides tools for analysing how the meaning of talk/text/objects shift over time and space. Such an approach has been adapted by Murdoch,^23^ to facilitate detailed investigation of complex healthcare interventions across multiple contextual levels and which is congruent with the socioecological model of behaviour. Four elements of context (see figure 2) will be investigated to capture variation in adoption, delivery and maintenance as well as responses to the intervention, affecting both reach and fidelity, which are likely to be important factors in outcome differences.

To situate the PACK Child intervention within this theoretical framework, we will develop a logic model, which first sets out contextual determinants of current paediatric illness management in South Africa, and second, how the PACK Child intervention components function to tackle these determinants to improve outcomes in the management of children.

### Overall design

The study will use a mixed method approach including quantitative and qualitative data collection methods in all primary care facilities. The process evaluation methods for each objective are described in table 1, including how each method maps onto different contextual levels. Quantitative methods include training attendance logs and staff questionnaires. Qualitative methods include observations of training sessions, semistructured interviews with parents/carers, individual or group interviews with staff and ethnographic observations of consultations and non-clinical areas.

**Table 1:** Objectives, contextual features and data collection for PACK Child process evaluation

Objective	Contextual level/feature	Data collection
(1) To determine the organisational and social context of PACK Child training as it is delivered and embedded into routine practice	Macro National policy and service protocols; broader context of South African healthcare system and role of primary care; funding and regulatory arrangements for primary care clinical practice; discourses of healthcare (eg, widely circulated discourses on management of paediatric chronic illnesses) Meso Local health area/facility policies or service protocols on management and treatment of patients; geographic and demographic profile of clinics; the main social actors involved in determining intervention delivery (ie, healthcare staff, patients, managers, policy makers) and the different sites and scenes which impact on how PACK is adapted and delivered (eg, local area management meetings, translation of PACK materials).	Macro Interviews with approximately 10 national and provincial managers and policymakers on structural interventions to facilitate PACK Child implementation. Documentary analysis of how guide/stationery integrate together and structure paediatric care. (i) PACK Child guides, (ii) IMCI guides, (iii) Integrated Clinical Stationery. Meso Training sessions’ attendance logs, including numbers and staff profiles. One day of observation of non-clinical areas per facility, such as waiting rooms and receptionists’ areas, to understand clinic organisation and deployment of staff, procedures and protocols which may impact on the implementation of PACK Child. One semistructured interview with the manager at each facility about organisational processes impacting on PACK Child.
(2) To determine the fidelity of intervention delivery	Micro Main (ie, training sessions, consultations) activities and subsidiary activities (eg, additional training/meetings, receptionist screening of patients) of intervention delivery and interactional arrangements of each activity.	Staff questionnaire on views and use of PACK Child during and at end of study. On average, 10 clinical staff per facility will be asked about number of training sessions attended and frequency of using PACK Child. One manager questionnaire per facility on numbers of staff, clinical rooms, scheduling and location of training sessions. Observation of training sessions at each site. Focus on delivery, response and points of difficulty. In Phase 1, observations of all eight training sessions will be conducted. This will inform theoretical sampling of observations of training in Phases 2 and 3. Up to three observations per facility will be conducted in Phases 2 and 3. Ethnographic observation of child consultations. With consent from both staff and carer/child, a researcher will observe and audio-record consultations to understand dosage and intensity of PACK Child integration into routine practice. In Phase 1, up to two observations will be conducted after each training session (16 in total) to develop the theoretical sampling frame for Phases 2 and 3. In Phases 2 and 3, up to four observations per facility will be conducted. At the end of Phase 3, we will also return to the Phase 1 clinic to observe up to four consultations to obtain insight into sustainability of PACK Child.
(3) To determine staff perspectives of delivery and participation in the training intervention	Perspectives on meso challenges and micro challenges of intervention delivery.	Staff questionnaire (average 10 per site—same questionnaire as detailed in Objective 2) on views and use of PACK Child including ease of use, impact on quality of care, management of patients, time management, prescribing and confidence in using PACK Child. Interviews with consenting facility trainers and two trained staff delivering intervention per facility at the completion of training. Interviews will explore facility context, views of training, barriers and facilitators of delivery, integration of PACK Child with IMCI during consultations, effect on workflow, clinical competency and interoperability of PACK Adult and PACK Child.
(4) To determine carer’s (and children as appropriate) perspectives of how PACK Child consultations meet carer and child needs	Perspectives on responses to interventions and experience of condition.	1. Interviews with carers and child (as appropriate). Carer’s/children from each facility will be asked to consent to interviews during implementation to understand their experiences and perceptions of their child’s health, previous contacts with primary and secondary healthcare and to assess their response to PACK Child within individual consultations. We will work with nurses in the facility to identify and approach suitable participants. Carers will also be asked to consent to observation of consultations. For a subsample of carers/children, we will therefore have both interview and ethnographic observational data. In Phase 1, up to two interviews will be conducted after each training session (16 in total) to develop the theoretical sampling frame for Phases 2 and 3. In Phases 2 and 3, up to four interviews will be conducted per facility.
(5) To determine how to optimise delivery of PACK Child within routine practice (6) To determine which components of the PACK Child intervention affect which change in healthcare processes and outcomes (7) Barriers to and facilitators of change through implementation of PACK Child guide in primary care	Analysis of the relationship between intervention components and macro-contextual, meso-contextual, micro-contextual features. This will enable the development of theoretical propositions of how to optimise delivery within routine practice to produce different outcomes.	Qualitative data will be triangulated to examine how PACK Child intervention interacts with macro-contextual, meso-contextual and micro-contextual features to affect which change. Development of theoretical propositions of how adjusting contextual features may bring about different outcomes. Quantitative and qualitative analyses will be integrated to produce an overall interpretation of implementation and theoretical fidelity of PACK Child. Findings will be shared with clinicians, managers and policymakers within three focus groups at end of Phase 3 to understand perspectives of how best to optimise delivery, barriers and facilitators of change.

IMCI, Integrated Management of Childhood Illness; PACK, Practical Approach to Care Kit.

Data collected from sites will provide a ‘thick description’,^24^ of how the intervention was delivered, maintained and experienced by staff and patients. The data will also offer explanations for observed variation between sites and detailed insight into the interaction between different contextual features and components of intervention implementation.

### Research setting

The setting for this pilot and process evaluation will be in 10 public-sector primary care clinics within four subdistricts (Southern Western Sub-structure, Klipfontein Mitchell’s Plain Sub-structure, Cape Winelands, South Peninsula), serving impoverished urban and rural communities in the Western Cape, South Africa. Phase 1 will take place in a single facility, Phase 2 will cover an additional three facilities and Phase 3 will cover a further six facilities. A KTU trainer, who has extensive clinical experience with children, both as an IMCI nurse and trainer (MS), will directly train facility staff onsite during the first two phases and will train facility trainers to train the remaining six facilities in Phase 3, using the same model used extensively for PACK Adult.

The proposed facilities were purposively selected by the Western Cape Health Department’s People Development Centre, which oversees training and upskilling of public sector healthcare workers in the Western Cape. Study sites were selected to provide maximum variation of primary care delivery, and exposure across urban and rural settings, municipal and provincial government. Factors considered important for observing variation included whether clinics were Ideal Clinic sites (an initiative to improve quality of primary healthcare),^25^ had IMCI-trained nurses, had varying levels of PACK Adult training coverage and were Integrated Clinical Stationery pilot sites (with checklist-enhanced child health records).

### Study population

The participants included in this study will be children and their parents or carers; staff working at the selected clinics, which includes nurses, doctors and other administrative staff; nurse educators delivering the training; managers of paediatric health services and policymakers.

To be eligible for inclusion, nurses and doctors will need to receive PACK Child training and carers and children will need to be receiving paediatric services at the selected clinics. Children will need to be aged 0–13 years to receive paediatric services. Policymakers will need to be responsible for delivery of primary care in South Africa.

### Sampling

Purposive sampling will be used in Phase 1 to select and recruit policymakers, staff, carers and children. Findings from the analysis of Phase 1 qualitative observation and interview data (eg, children’s presenting conditions or challenging aspects of using the PACK Child guide) will be used to inform theoretical sampling^26^ of staff, carers and children and timing of data collection in Phases 2 and 3.

*Staff:* Managers and nurses and doctors who have received training, at each primary care facility will be approached to participate in individual or group interviews, and observations and recording of consultations.

*Parent/Carer/Child:* Purposive sampling of children in Phase 1 will be informed by diversity of conditions, level of deprivation and age of child. We will work with clinic nurses to identify and approach suitable participants in clinic waiting room areas. Carers and children will be asked to consent to the process evaluation on the day they attend the clinic.

*Policymakers:* Key informants at provincial and national level involved in the management of child health services or supporting policymaking will be identified and invited by email to participate in a face-to-face or telephonic interview. Potential informants could include the provincial head of paediatric services, a paediatric specialist in the national department of health and representatives from the WHO and Unicef country office in South Africa.

### Ethical considerations

The key ethical principles of voluntary and informed participation, confidentiality and safety of participants will be used in all researcher and participant interactions. Written consent for interviews and observations will be obtained from all policy stakeholders, facility managers, staff and carers. Children over 7 years old will be required to give assent to their participation. Facility managers will provide consent for observations of training sessions and non-clinical areas. All participants will be provided with written information about the research, informed that their participation is voluntary and that they may withdraw from participation at any time.

Safety of all data will be ensured by: (1) encrypting all transcriptions with a password protected code; (2) all data uploaded to Google Drive will be encrypted and only accessible by the research team; (3) all personal information of participants will be kept separately from all transcripts and each participant given a participant identification number; (4) on completion of the study all files on the Google Drive will be deleted.

### Data analysis

#### Qualitative data

To empirically observe the interaction between different contextual features and intervention implementation, we will first examine how PACK Child is delivered within facilities. Second, we will investigate where implementation of PACK Child triggers disruptions to routine practice or where delivery of PACK Child itself is disrupted or breaks down. Such an approach provides ‘telling cases’,^27^ exposing wider social forces structuring intervention delivery *at the point of delivery*, relations which are otherwise hidden from view. To help identify and analyse disruptions, we will track how the PACK intervention is transformed across different contextual levels. Disruptions are likely to be located within debates between participants during training sessions; problems incorporating PACK Child into clinic processes; reported challenges/problems of delivery or resistance by staff, caregivers and children; misunderstandings about PACK Child shown in interactions between trainers and staff, staff and patients; difficulties nurses demonstrate when using PACK Child and IMCI together during consultations and health system obstacles such as medicine stock outs or lack of essential equipment. This analytical work will be fundamental in generating hypotheses about the relationship between context, mechanism and outcomes; key difficulties in adoption, delivery and maintenance and importantly, how to optimise the PACK Child intervention for wider implementation.

*Observations of Training Sessions:* We shall conduct a content analysis of field notes of observations to identify difficulties in delivery, in particular, any tensions between the content of PACK Child and existing practice within each clinic.

*Interviews and focus groups:* All interviews and focus groups will be transcribed verbatim and thematically analysed using NVivo software. This will provide detailed perspectives of the process and content of implementing PACK Child. We will then develop a coding scheme for the identified themes and structure according to different contextual levels and mechanisms of impact. A constant comparison approach will be adopted, working iteratively between data obtained from different interviewees within and between clinics as well as perspectives from higher contextual levels (policymakers and managers).

*Ethnographic observations:* Field notes will be analysed thematically in the first instance to provide a description of process and content involved in adapting and delivering PACK Child, to identify issues of fidelity over time. Audio recordings of consultations will be transcribed verbatim with a subsample transcribed using conversation analytic conventions,^28^ which will provide detailed evidence of how staff’s use of PACK Child is negotiated within interactions with carers and children. We will then analyse the ethnographic data to provide a ‘thick description’,^24^ of how PACK Child is organised and implemented within the specific social historical contexts and to identify moments of difficulty, where implementation is disrupted or breaks down.

#### Quantitative data

The quantitative data (health worker questionnaire and training attendance logs) will be subject to descriptive statistical analysis to build a picture of how PACK Child was used and perceived within and across all 10 primary care facilities and the health systems context to enhance or hinder implementation. This will enable individual study sites to be situated in the range of implementation fidelity and perceptions of delivery across all facilities.

### Data synthesis

**Figure 3:**
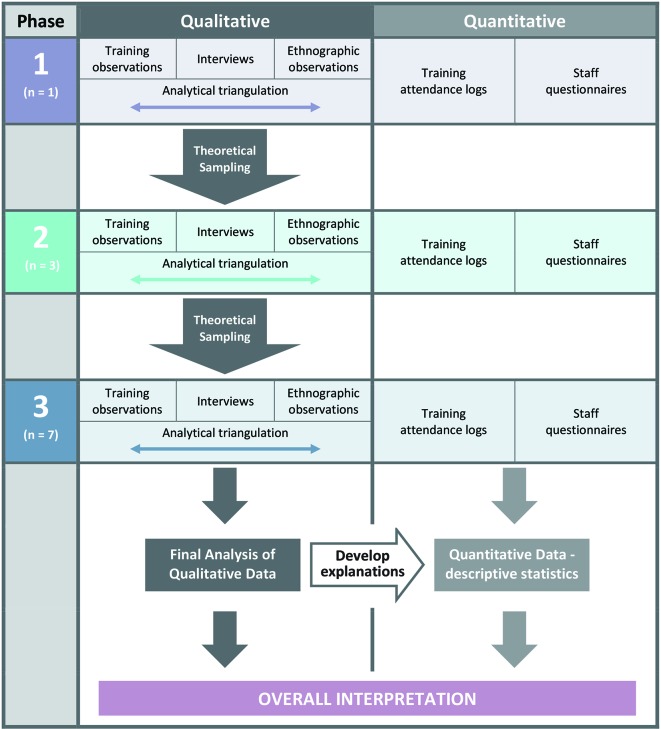
Theoretical sampling and synthesis of process evaluation data.

The analysis of qualitative data will be iterative, moving between data collection and analysis to test emerging theories (see figure 3). It may, for example, emerge that some health workers have expectations about PACK Child which shape their experience and use of the intervention, and this may require deeper exploration. The analysis of the ethnographic data will therefore require knowledge from health worker interviews to compare how reported experience relates to actual implementation of PACK Child. Care will be taken to identify and follow up deviant cases which do not fit into emerging theories. Quantitative and qualitative data will be triangulated,^29^ to understand how different types of evidence enhance the overall interpretation of how PACK Child is delivered and to develop possible explanations for implementation variation.

By setting the delivery of the PACK Child intervention within a macro-contextual, meso-contextual and micro-contextual framework, we will be able to make the transition from the identification of routines and patterns of the use of PACK in specific facilities, to theoretical explanations of how different structural relations and mechanisms organise moments of delivery, which then impact on specific outcomes. Instances of disruption to delivery at a micro level offer such cases, exposing how the active ingredients of PACK Child are organised and operate within the specific social historical contexts of implementation. Identifying ‘telling cases’,^27^ will facilitate generalisable inferences and predictions on how to best to scale-up the PACK Child intervention.

In drawing case comparisons across clinics, we will develop hypotheses about why the intervention is linked to outcomes which we can test out in a future pragmatic trial. This may lead us to identify factors which are plausibly and/or consistently related to successful or unsuccessful delivery of the components of the intervention. Emerging theories and the relationship of the data to the conceptual literature underpinning the intervention will be discussed and refined at research team meetings throughout the research.

## Conclusion

This paper reports the design and methods for the planned process evaluation of the pilot and feasibility study of PACK Child providing the opportunity to document implementation and collaboratively refine the programme prior to testing in a pragmatic trial or wider implementation. The process evaluation protocol conforms to recommendations intended to facilitate standardisation of process evaluation design and reporting,^16 17^ in order that synthesis of results of similar studies may become possible in future.
